# TFE3 and HIF1α regulates the expression of SHMT2 isoforms via alternative promoter utilization in ovarian cancer cells

**DOI:** 10.1038/s41419-025-07445-y

**Published:** 2025-03-17

**Authors:** Si-Qi Wang, Ning Liu, Qi Zhang, Bai-Qiang Li, Fu-Ying Zhao, Chao Li, Hua-Qin Wang, Chuan Liu

**Affiliations:** 1 Department of Biochemistry & Molecular Biology, China Medical University, Shenyang, 110026 China; 2 The Department of Radiation Oncology, The First Hospital of China Medical University, Shenyang, 110001 China; 3 Department of Obstetrics and Gynecology, Shengjing Hospital of China Medical University, Shenyang, 110004 China; 4 Criminal Investigation Police University of China, Shenyang, 110006 China

**Keywords:** Ovarian cancer, Drug regulation

## Abstract

Ovarian cancer ranks first lethally among gynecological malignancies. Platinum-based chemotherapy constitutes the first-line therapeutic regime. However, primary or acquired resistance seriously affects the survival rate of patients with ovarian cancer. Serine hydroxy methyltransferase (SHMT) catalyzes conversion of serine to glycine and is responsible for production of S-adenosylmethionine (SAM) for methylation. There are cytosolic SHMT1 and mitochondrial SHMT2 in human. Alternative promoter usage is a proteome-expanding mechanism that allows multiple pre-mRNAs to be transcribed from a single gene. The current study demonstrated that cisplatin-sensitive and cisplatin-resistant ovarian cancer cells expressed discrete SHMT2 isoforms, which was ascribed to the selective utilization of *SHMT2* alternative promoters. SHMT2 isoforms exerted somewhat paradoxical roles in ovarian cancer cells, with tumor-suppressive role of isoform 1, and tumor-promotive role of isoform 3. In addition, the current study demonstrated that SHMT2 alternative promoter usage mediated by HIF1α and TFE3 might represent adaptive response of ovarian cancer cells to metabolic stress. Collectively, regulation of SHMT2 isoform expression via alternative promoter usage by transcription factors HIF1α and TFE3 provides a novel basis and potential drug targets for the clinical treatment of platin-resistant ovarian cancer.

## Introduction

Ovarian cancer is one of the most common and lethal gynecological malignancy. Due to the concealment of the incidence and the lack of perfect early diagnosis methods, about 70% of the patients were diagnosed at an advanced stage [1–3]. Although a certain clinical effect has been obtained based on the generally treated by tumor cell reduction surgery combined with platinum-based chemotherapy, ovarian cancer patients still may have a primary or acquired resistance to chemotherapeutic drugs, especially platinum drugs in first-line chemotherapy, and it seriously affects the quality of life and survival rate of patients [4–6]. However, there is no specific drug for clinical treatment of recurrent platinum-resistant ovarian cancer. Therefore, to improve the sensitivity of ovarian cancer chemotherapy and reverse platinum resistance is an urgent problem to be solved in the treatment of ovarian cancer.

Glycolysis is abnormally activated in tumor cells and becomes an important source for energy production [7]. Serine synthesis pathway as a branch of glycolysis, which contributes to nucleotide synthesis, methylation reactions and generation of NADPH for redox homeostasis, has received more and more attention. As a non-essential amino acid, normal cells can rely on endogenous synthesis to satisfy the proliferation needs, while tumor cells also need exogenous intake due to accelerated proliferation and enhanced metabolism [7]. Enzymes responsible for the serine synthesis pathway are highly expressed in various cancers, including colorectal cancer, breast cancer, and hepatocellular carcinoma. Serine is converted into glycine and a tetrahydrofolate by serine hydroxy methyltransferase (SHMT), SHMT1 mainly exists in cytosolic and SHMT2 mainly exists in mitochondrial [8].

SHMT2 is a key mitochondrial enzyme in serine catabolism that converts serine to glycine and produces a one-carbon unit that generates S-adenosylmethionine (SAM) for methylation. Recent studies have shown that SHMT2 is significantly overexpressed in a variety of tumors and is associated with poor prognosis [9–13]. Increased serine catabolism supports malignant growth through diverse mechanisms [14]. However, the functions and underlying mechanisms of SHMT2 in ovarian cancer remain rarely studied.

Alternative promoter refers to the variable region corresponding to the initial start site of a specific gene transcript. It was found that about 36 and 40% of protein coding genes in human and mouse genomes respectively have variable promoters [15]. The selection of alternative promoters greatly increases the diversity of transcriptome, however, the research on the biological function and molecular mechanism of alternative promoters is still very scarce. The current study reported that HIF1α and TFE3 were implicated in the utilization of *SHMT2* alternative promoter, resulting in expression of SHMT2 isoforms in cisplatin-resistant ovarian cancer cells under metabolic stress. Our study verified complicated implication of SHMT2 isoforms in ovarian cancer cells.

## Method and materials

### Clinical samples

36 patients with ovarian cancer who underwent surgical resection in Shengjing Hospital of China Medical University from June 2014 to July 2017 were enrolled in this project. None of the patients had received radiotherapy or chemotherapy before the surgery. The patients were divided into platinum-sensitive group (24 cases) and platinum-resistant group (12 cases) according to the recurrence within 6 months after the first cisplatin chemotherapy. The collected tissues were frozen in liquid nitrogen and stored in a refrigerator −80 °C for further analysis. The program was approved by the Institutional Review Board of China Medical University without the informed consent of patients or their families.

### Proteomic assay

The sample was grinded with liquid nitrogen into cell powder and then transferred to a 5-mL centrifuge tube. After that, four volumes of lysis buffer (8 M urea, 1% protease inhibitor cocktail) were added to the cell powder, followed by sonication three minutes on ice using a high intensity ultrasonic processor (Scientz). The remaining debris was removed by centrifugation at 12,000 × *g* at 4 °C for 10 min. Finally, the supernatant was collected and the protein concentration was determined with BCA kit according to the manufacturer’s instructions. Then sample was slowly added to the final concentration of 20% (m/v) TCA to precipitate protein, then vortexed to mix and incubated for 2 h at 4 °C. The precipitate was collected by centrifugation at 4500 × *g* for 5 min at 4 °C. The precipitated protein was washed with pre-cooled acetone for 3 times and dried for 1 min. The protein sample was then redissolved in 200 mM TEAB and ultrasonically dispersed. Trypsin was added at 1:50 trypsin-to-protein mass ratio for the first digestion overnight. The sample was reduced with 5 mM dithiothreitol for 30 min at 56 °C and alkylated with 11 mM iodoacetamide for 15 min at room temperature in darkness. Finally, the peptides were desalted by Strata X SPE column. The raw data has been uploaded to iProx platform.

### LC-MS analysis

The tryptic peptides were dissolved in solvent A, directly loaded onto a home-made reversed-phase analytical column (25-cm length, 100 μm i.d.). The mobile phase consisted of solvent A (0.1% formic acid, 2% acetonitrile/in water) and solvent B (0.1% formic acid in acetonitrile). Peptides were separated with following gradient: 0–14 min, 6–24%B;14–16 min, 24–35%B; 16–18 min, 35–90%B; 18–20 min, 90%B, and all at a constant flow rate of 500 nl/minon an Easy-nLC1000 UHPLC system (Bruker Daltonics). The peptides were subjected to capillary source followed by the timsTOF Pro mass spectrometry. The electrospray voltage applied was 1.75 kV. Precursors and fragments were analyzed at the TOF detector. The timsTOF Pro was operated in data independent parallel accumulation serial fragmentation (dia-PASEF) mode. The full MS scan was set as 300–1500 (MS/MS scan range) and 20PASEF (MS/MS mode) -MS/MS scans were acquired per cycle. The MS/MS scan range was set as 400–850 and isolation window was set as 7 m/z.

### Clinicopathological characteristics analysis and survival analysis

The correlation between SHMT2 expression and clinicopathological characteristics was explored using UALCAN (http://ualcan.path.uab.edu/), a web portal to analyze the relative expression of the desired gene(s) in tumor and normal samples, and association with clinicopathological characteristics of the patients such as cancer stage, tumor grade. We collected survival information of 1440 ovarian cancer patients from GEO, EGA, and TCGA databases and used them to examine the effect of SHMT2 on the prognosis of OV using Kaplan–Meier curve 18 and GEPIA databases. The examination probe ID used for SHMT2 was 227198_at. The log-rank P-value and hazard ratio (HR) with 95% confidence intervals (CI) were analyzed.

### Single-cell RNA sequencing analysis

The single-cell RNA-sequencing of ovarian cancer were downloaded from Gene Expression Omnibus (GEO, https://www.ncbi.nlm.nih.gov/geo/, GSE138794) and analyzed using R package “Seurat 4.1.0”. Method “UMAP” was applied for the visualization of different cell clusters.

### Cell culture

The human ovarian cancer cell lines SKOV3 and A2780, as well as their cisplatin-resistant counterparts SKOV3/DDP and A2780/DDP were provided by Shanghai Genechem Co., Ltd. Cells were maintained in RPMI-1640 (Life Technologies, USA) with 10% fetal bovine serum (FBS, Sigma, USA) and penicillin (100 IU/ml, Sigma, USA) and streptomycin (100 µg/ml, Sigma, USA) and the cells were maintained at 37 °C with 5% CO2.

Four groups cell culture condition were used in this experiment: normal culture condition: 20%O2 + 25 mM Glu, hypoxia culture condition:1%O2 + 25 mM Glu, low Glu culture condition: 20%O2 + 2.5 mM Glu, hypoxia + low Glu culture condition: 1%O2 + 2.5 mM Glu.

### Western blot and immunoprecipitation

Total cellular proteins were extracted using the lysis buffer containing Tris-HCl, 150 mM NaCl, 2 mM EDTA, 1% Triton-X100, and a protease inhibitor cocktail (Sigma-Aldrich, St. Louis, MO), and the extracted protein was quantified by the BCA protein assay kit. 20 μg total protein was separated with 10% SDS-PAGE and transferred to PVDF membrane (Millipore Corporation, Billerica, MA). The immunoprecipitants were treated with various antibodies, incubated overnight in 4 °C, washed three times with lysis buffer, and analyzed using Western blot. The following primary antibodies were used in our manuscript: Tubulin(1:1000, Abcam, ab7291), COL1A1(1:1000, Abnova, A01), a-SMA(1:1000, Sigma-Aldrich, A5228), EpCAM (1:1000, MCE, YA772), CD44(1:1000, Abcam, ab254530), CD133(1:1000, MCE, HY-80054),TFE3 (1:1000, Sigma-Aldrich, 354R-1), HIF1α(1:1000, Sigma-Aldrich, H6536) and XBP1(1:1000, Novus biologicals, NBP1-77681).

### Knockdown of SHMT2 by CRISPR-Cas9

Lentivirus infection experiments were performed to knockdown SHMT2 stably. Lentivirus was purchased from Nanjing Jikeyin Company. 1 × 10^5^ cells/well were inoculated in 6-well plates and cultured for 24 h. The virus solution was added into the cell culture medium and cultured for 8 h, then the culture medium was replaced with a normal medium containing 1 μg/ mL purinomycin for 48 h, and the protein was extracted and verified by Western blot.

### Cell Counting Kit 8 (CCK-8) assay

CCK8 (Dojindo Laboratories) assays was performed by exposing cells to different concentrations of cisplatin for 24 h. 10 μL diluted CCK-8 solution was added to each well. Cells were incubated at 37 °C for 4 h, and the absorbance at 450 nm was determined with a microplate reader.

### Sphere formation assay

Logarithmic growth phase cells were harvested and resuspended with 20 mg/ mL EGF (Sigma-Aldrich, St. Louis, MO, USA), 5 μg/ mL insulin (Sigma-Aldrich) and 2% B27 (Invitrogen, Carlesbad, CA, USA) in serum-free DMEM/F12 medium. The cells were inoculated in 6-well plates, the medium was changed every 3 days. The cell morphology was observed, and the images were taken under the microscope. The number of spheres was counted according to the images.

### Nude mouse xenograft experiments

BALB/c-nu/nu mice (4–5 weeks old, female) were purchased from Liaoning Changsheng Biotechnology Co., Ltd. All animal procedures were approved by and compiled with the guidelines of the Institutional Animal Care Committee of China Medical University. Mice were random subcutaneously inoculated with the specified number of viable SKOV3/DDP and A2780/DDP cells. The status of the mice and tumor formation were observed over time, and the mice were sacrificed after 28 days. The subcutaneous tumors were removed and photographed. The subcutaneous tumors were removed and photographed. Statistical analysis of tumor formation in the nude mice was performed by extreme limiting dilution analysis (ELDA) software (http://bioinf.wehi.edu.au/software/elda/).

### Immunofluorescence (IF) staining

Cells were fixed with 4% paraformaldehyde for 15 min and permeabilized with 0.1% Triton X-100 in PBS. The cells were blocked with PBS containing 1% bovine serum albumin for 1 h at room temperature. Immunostaining was performed using the primary antibody against TFE3 and HIF1α and fluorescent secondary antibody. Finally, cells were mounted and visualized using the Cytation 5 Cell Imaging Multi-Mode Reader (BioTek Instruments).

### Chromatin immunoprecipitation (ChIP) assay

ChIP was performed according to the manual using the ChIP^TM^ assay (Merck Millipore, Billerica, Massachusetts). 1 × 10^7^ cells were fixed with 1% formaldehyde for 10 min and quenched with 0.125 M glycine for 10 min. Cells were washed and resuspended sequentially in three lysis buffers to isolate chromatin. Chromatin was sonicated and incubated overnight at 4 °C with 10 µg of antibody for the indicated proteins and 50 µl of Protein G magnetic beads. Magnetic beads were the washed and followed by DNA purification. The ChIP and input DNAs were measured by quantitative PCR qPCR using intergenic regions as negative controls.

### Proximity ligation assay (PLA)

Cells were seeded on coverslips in 12-well plates during 48 h with DMSO (Sigma-Aldrich) or indicated molecules (10 µM, DMSO < 0.001%). After 48 h of culture, cells were fixed using 4% PBS-PFA, permeabilized using 100% chilled methanol and incubated at 4 °C with TFE3 (1:1000, Sigma-Aldrich, 354R-1) or HIF1α(1:1000, Sigma-Aldrich,H6536) antibodies. Duolink® experiments were performed according to the manufacturer’s protocol (Sigma-Aldrich). Microscopy images were obtained by an Axio Imager 2 (Carl Zeiss Microscopy GmbH) and images were acquired using AxioCam MRm CCD camera (Carl Zeiss GmbH, ×63 oil objective). The Spot detector plugin of ICY software was used, and statistical analysis was performed with GraphPad Prism (Unpaired Man and Whitney, at least 100 cells counted for each condition).

### Untargeted metabolomics assay

The sample stored at −80 °C refrigerator was thawed on ice and vortexed for 10 s. 50 μL of sample and 300 μL of extraction solution (ACN: Methanol = 1:4, V/V) containing internal standards were added into a 2 mL microcentrifuge tube. The sample was vortexed for 3 min and then centrifuged at 12000 rpm for 10 min (4 °C). 200 μL of the supernatant was collected and placed in −20 °C for 30 min, and then centrifuged at 12000 rpm for 3 min (4 °C). A 180 μL aliquots of supernatant were transferred for LC-MS analysis. The mass spectrometer was operated in ESI positive and negative modes with voltages of 2500 V or 1500 V. The gas temperature and sheath temperature was both set at 325 °C, with a gas flow rate of 8 mL/min. The fragmentor was 135 V, sheath flow was 11 ml/min and nebulizer were 40 psi in positive or negative modes.

The original data file acquihired by LC-MS was converted into mzML format by ProteoWizard software. Peak extraction, peak alignment and retention time correction were respectively performed by XCMS program. The “SVR” method was used to correct the peak area. The peaks with detection rate lower than 50% in each group of samples were discarded. After that, metabolic identification information was obtained by searching the laboratory’s self-built database, integrated public database, AI database and metDNA.

### Statistical analysis

ANOVA and post hoc Dunnett’s test were used to analyze the statistical significance in the most experiments, the significant difference was defined as *p* < 0.05. All experiments were repeated 3 times, and data were expressed as mean ± SD (standard deviation) of representative experiments.

## Results

### Distinct expression of SHMT2 isoform 1 and 3 in cisplatin-sensitive and cisplatin-resistant ovarian cancer

**Fig. 1:**
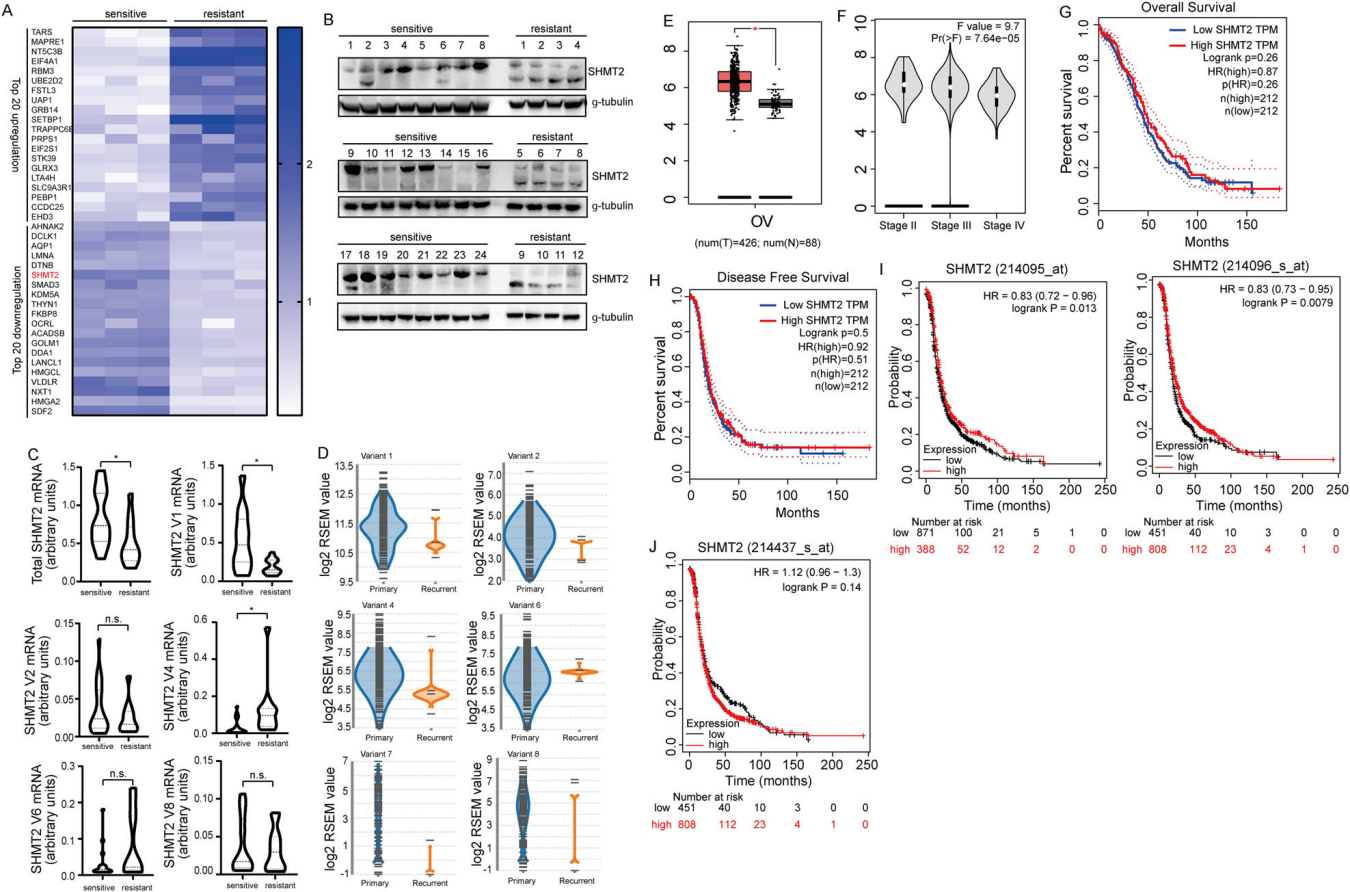
Cisplatin-sensitive and cisplatin-resistant ovarian cancer mainly express SHMT2 isoform 1 and 3, respectively. **A** Top 20 differentially expressed proteins between cisplatin-sensitive and resistant pathological tissue in ovarian cancer. **B** Western Blot showed the presence of SHMT2 and SHM2a isoforms in ovarian cancer. **C** RT-PCR showed that mRNA expression in real-time in different variants expression between cisplatin sensitive and resistant ovarian cancer tissue. **D** Different variants expression between cisplatin sensitive and resistant ovarian cancer in bulk database. **E**–**H** Different expression of SHMT2 in GEPIA database. **I**, **J** Kaplan–Meier curves revealing overall survival of SHMT2s’ expression according to different Affymetrix microarrays detected.

To clarify the mechanisms underlying cisplatin resistance in ovarian cancer, quantitative proteomics analysis was performed to globally screen the potential molecular targets regulated by cisplatin resistance. We conducted the mixture of three samples for each of the 9 cisplatin-sensitive and 9 cisplatin-resistant ovarian cancer tissues and performed quantitative proteomics analysis on the combined samples. SHMT2 was identified to be downregulated in the cisplatin-resistant ovarian cancer tissues (Fig. 1A). Expression of SHMT2 was further analyzed using 24 cisplatin-sensitive and 12 cisplatin-resistant ovarian cancer tissues and confirmed the downregulation of SHMT2 in cisplatin-resistant ovarian cancer tissues (Fig. 1B). Two SHMT2 isoforms were identified in ovarian cancer tissues, the smaller isoform was upregulated, even though downregulation of total SHMT2 in cisplatin-resistant tissues (Fig. 1B). Real-time RT-PCR demonstrated that the total SHMT2 mRNA expression was significantly decreased in cisplatin-resistant compared to the cisplatin-sensitive ovarian cancer tissues (Fig. 1C). There are multiple SHMT2 variants, the individual variant expression was then studied and found that variant 1 and variant 4 were significantly decreased and increased in the cisplatin-resistant tissues, which encode isoform 1 and isoform 3 of SHMT2 respectively (Fig. 1C). With regards to other variants, there were no significant difference in the cisplatin-sensitive and cisplatin-resistant ovarian cancer tissues (Fig. 1C). The online splicing data from TCGA database (tsvdb.com/index.html) also confirmed expression of multiple SHMT2 variants in ovarian cancer tissues (Fig. 1D). To further determine the potential importance of SHMT2 in clinical settings, we analyzed clinical outcomes from GEPIA OV samples. Compared with normal tissues, the expression level of SHMT2 was significantly increased (Fig. 1E). In addition, the expression of SHMT2 among Stage II, Stage III and Stage III group were significantly different (Fig. 1F). Kaplan–Meier curve showed that neither OS (Fig. 1G) nor PFS (Fig. 1H) were significantly associated with the mRNA expression level of SHMT2. To better understand the relevance of SHMT2 expression in OV patients, KM plotter was used to evaluate prognostic value based on Affymetrix microarrays. It is worth noting that the expression of SHMT2 was detected by different Affymetrix microarrays, the effect on prognosis was different (Fig. 1I, J). This might because different probes recognize different SHMT2 variants, and the different effects on prognosis suggest that different SHMT2 variants might have different biological behaviors, which may be related to cisplatin resistance.

### SHMT2 isoform 3 is expressed in cisplatin-resistant ovarian cancer under metabolic stress

**Fig. 2:**
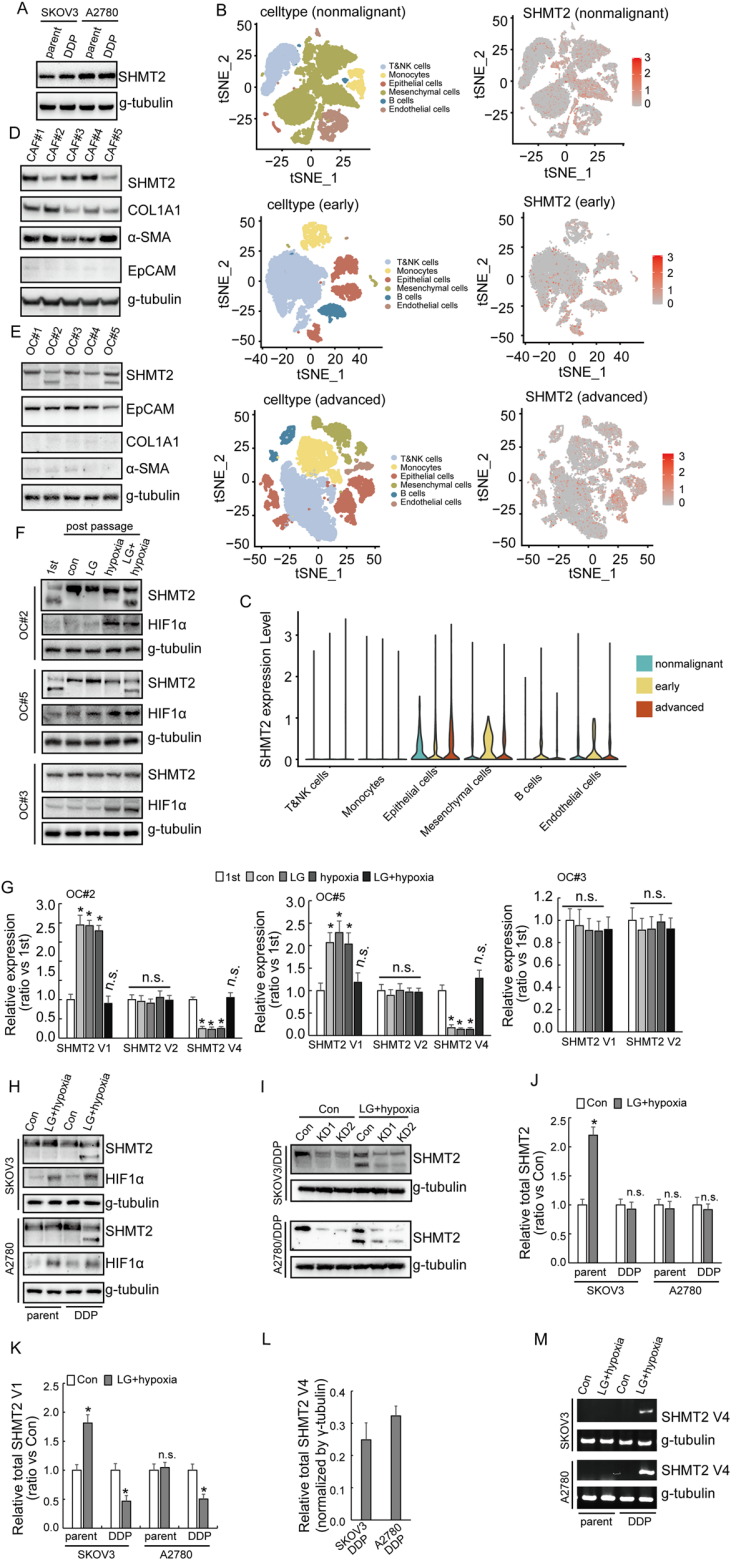
SHMT2 isoform 3 is expressed in cisplatin-resistant ovarian cancer under metabolic stress. **A** Western blotting of SHMT2 expression in cisplatin-sensitive (parental) and cisplatin-resistant (DDP) SKOV3 or A2780 cells. **B**, **C** Single-cell RNA sequencing of the expression of SHMT2 among different stages visualizing UMAP cell clusters. **D** Western blotting of SHMT2 expression in CAF extracted from patients with ovarian cancer. **E** Western blotting of SHMT2 expression in ovarian cancer cells extracted from patients. **F** Western blotting of SHMT2 expression in ovarian cancer cells extracted from patients under different situations. **G** RT-PCR showed the difference among different SHMT2 isomers under different situations. **H** Western blotting of SHMT2 expression in cisplatin-sensitive (parental) and cisplatin-resistant (DDP) SKOV3 or A2780 cells under different situations. **I** Western blotting of SHMT2 expression upon SHMT2 knockout in cisplatin-resistant (DDP) SKOV3 or A2780 cells under different situations. **J**–**L** RT-PCR showed SHMT2, SHMT2 V1 and SHMT2 V4 in cisplatin-sensitive (parental) and cisplatin-resistant (DDP) SKOV3 or A2780 cells under different situations. **M** Agarose gel electrophoresis showed the expression of SHMT2 V4 in cisplatin-sensitive (parental) and cisplatin-resistant (DDP) SKOV3 or A2780 cells under different situations.

To further explore the role of SHMT2 isoforms in the process of cisplatin resistance, SHMT2 expression was studied using paired cisplatin-resistant and parental SKOV3 and A2780 cells. SHMT2 expression did not show significant difference between the cisplatin-resistant SKOV3 (SKOV3/DDP) and A2780 (A2780/DDP) cells compared with their respective parental cells (Fig. 2A). In addition, only single band representing SHMT2 isoform 1 was observed in ovarian cancer cell lines (Fig. 2A). Single-cell RNA sequencing analysis showed that SHMT2 was mainly expressed in epithelial and mesenchymal cells, in addition, SHMT2 also expressed in endothelial cells (Fig. 2B,C). Next, cancer-associated fibroblast (CAF) and ovarian cancer (OC) cells were extracted from fresh ovarian cancer tissues, Western blot demonstrated that CAF expressed SHMT2 isoform 1, while some OC cells expressed SHMT2 isoform 3 (Fig. 2D, E), suggesting that the expression of SHMT2 isoform 3 might be mainly derived from ovarian tumor epithelial cells. SHMT2 isoform 3 could be detected in some primary OC cells at the beginning, but with continuous cell culture, SHMT2 isoform 3 could not be detected. However, SHMT2 isoform 3 could be detected again under the conditions of hypoglycemia and hypoxia (Fig. 2F). Real-time RT-PCR showed that variant 1 decreased, while variant 4 decreased in OC#2 and OC#5 after 2–3 passages, culture under hypoglycemic and hypoxic condition recovered the expression of variant 4 (Fig. 2G). On the other hand, such fluctuation of expression of SHMT2 variants was not observed in OC#3, which did not express SHMT2 variant 4 originally (Fig. 2G). In the cisplatin-resistant SKOV3 (SKOV3/DDP) and A2780 (A2780/DDP) cells, SHMT2 isoform 3 could be detected in cisplatin-resistant SKOV3/DDP and A2780/DDP cells under hypoglycemic and hypoxic culture but could not be detected in their respective parental cells (Fig. 2H). SHMT2 isoform 1 and 3 were both downregulated when SHMT2 was knocked out under LG/hypoxia conditions (Fig. 2I). RT-PCR showed that hypoglycemic and hypoxic culture increased total and variant 1 SHMT2 expression in SKOV3 cells, while such induction was not observed in A2780 cells (Fig. 2J, K). SHMT2 variant 1 was consistently decreased (Fig. 2J), while SHMT2 variant 4 was consistently increased (Fig. 2L, M) in cisplatin-resistant cells cultured under hypoglycemic and hypoxic condition. In general, OC can express SHMT2 variant 4 only when metabolic stress occurs, that is, under a condition of low-glucose and hypoxic, thereby expressing the SHMT2 isoform 3.

### Different effects of SHMT2 knockout in cisplatin responsiveness in cisplatin-resistant and sensitive ovarian cancer cells

**Fig. 3:**
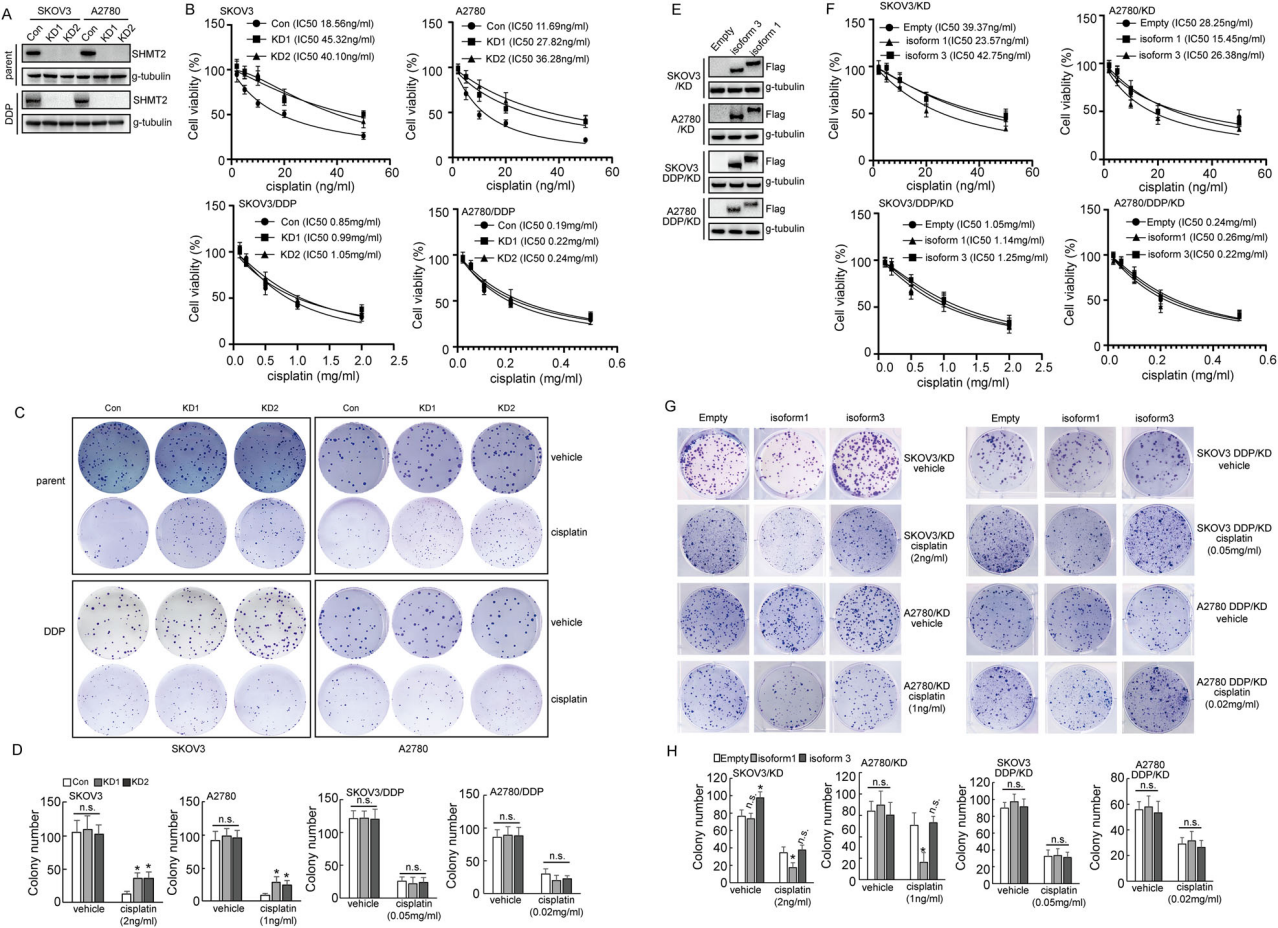
SHMT2 knockout had discrete effects on cisplatin responsiveness of cisplatin-resistant and sensitive ovarian cancer cells. **A** Western blot analysis of SHMT2 protein expression upon SHMT2 knockout in cisplatin-sensitive (parental) and cisplatin-resistant (DDP) SKOV3 or A2780 cells. **B** The quantification of cell viability of cisplatin-sensitive SKOV3 and A2780 cells treated with the indicated concentrations of cisplatin for 24 h, using CCK8 assays. **C** The quantification of cell viability of cisplatin- resistant (DDP) SKOV3 and A2780 cells were treated with the indicated concentrations of cisplatin for 24 h, cell viability was assessed using CCK8 assays. **D**, **E** Colony number shown upon SHMT2 knockout in cisplatin-sensitive (parental) and cisplatin-resistant (DDP) SKOV3 or A2780 cells. **F** Western blot analysis of SHMT2 and SHMT2a protein expression in cisplatin-sensitive (parental) and cisplatin-resistant (DDP) SKOV3 or A2780 cells. **G** The quantification of cell viability of cisplatin- resistant (DDP) SKOV3 and A2780 cells transduced with SHMT2 and SHMT2α overexpression sequences, which were treated with the indicated concentrations of cisplatin for 24 h, **H** Colony number shown upon SHMT2 and SHMT2α overexpression in cisplatin-sensitive (parental) and cisplatin-resistant (DDP) SKOV3 or A2780 cells.

In order to explore the role of SHMT2 in cisplatin resistance, SHMT2 was knocked out using CRISP/Cas9 system in SKOV3/DDP and A2780/DDP and their respective parental cells (Fig. 3A). Knockout of SHMT2 increased the survival of both SKOV3 and A2780 cells exposed to cisplatin treatment. However, in SKOV3/DDP and A2780/DDP cells knockout of SHMT2 did not show a significant difference in the viability of the cells after cisplatin exposure (Fig. 3B). Colony formation assay got a similar result. Knockout of SHMT2 increased the colony number of cisplatin sensitive cells, while there was no significant change in the resistant cells under cisplatin treatment (Fig. 3C, D).

To further understand the role of SHMT2 isoforms in cisplatin resistance, SHMT2 knockout cells were restored with SHMT2 isoform 1 and 3, respectively (Fig. 3E). Restoration of SHMT2 isoform 1, but not isoform 3, significantly decreased the cell viability (Fig. 3F) and colony formation (Fig. 3G, H) of SKOV3 and A2780 cells under cisplatin exposure. SHMT2 isoform 3 increased colony formation of SKOV3 cells, but not A2780 cells (Fig. 3G, H). Neither isoform 1 nor isoform 3 demonstrated any effects on viability and colony formation of SKOV3/DDP and A2780/DDP cells (Fig. 3F–H).

### Opposite roles of SHMT2 isoforms in maintenance of stem cell like features of ovarian cancer cells

**Fig. 4:**
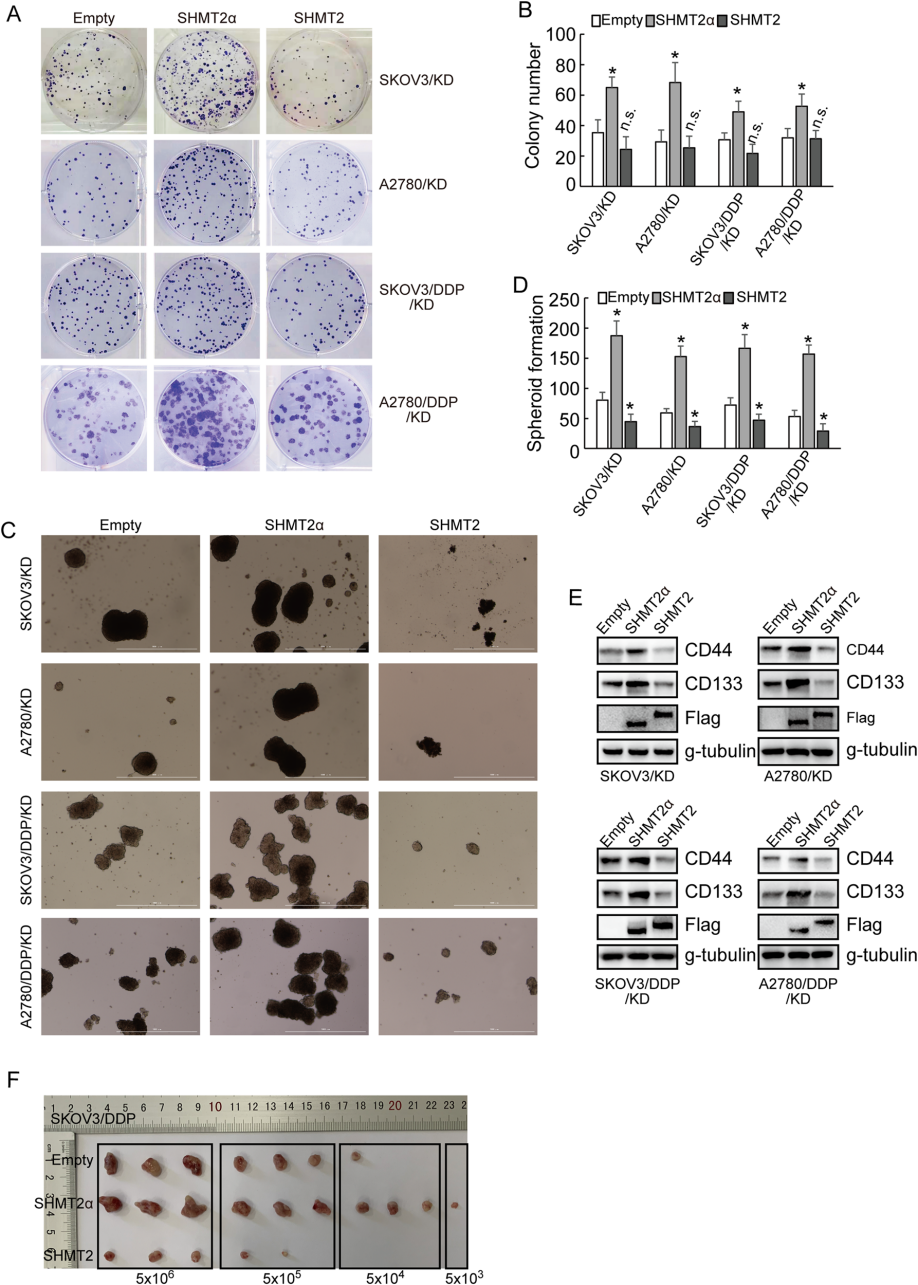
Opposite roles of SHMT2 isoforms in maintenance of stem cell like features of ovarian cancer cells. **A**, **B** Colony number shown upon SHMT2 and SHMT2α overexpression in cisplatin-sensitive (parental) and cisplatin-resistant (DDP) SKOV3 or A2780 cells under a low-glucose and hypoxic environment. **C**, **D** The quantification of tumor sphere formation of cisplatin-sensitive (parental) and cisplatin-resistant (DDP) SKOV3 or A2780 cells transduced upon SHMT2 and SHMT2α overexpression sequences under a low-glucose and hypoxic environment. **E** Western blot analysis of CD44 and CD133 protein expression upon SHMT2 and SHMT2α overexpression sequences under a low-glucose and hypoxic environment. **F** Image of the xenograft tumors dissected from nude mice (*n* = 3 in each group) was shown, and the ultimate weight and volume of the tumors were evaluated.

To distinguish the isoforms of SHMT2 more conveniently, we denominated SHMT2 isoform 3, which expressed solely in metabolic stress conditions, as SHMT2α. Next, in order to further understand the roles of SHMT2 isoforms, a low-glucose and hypoxic cell culture environment were constructed for the following experiments. SHMT2α, but not SHMT2, significantly promoted proliferative potential of both cisplatin-sensitive and cisplatin-resistant cells under low-glucose and hypoxic conditions according to the results of the clone formation assay (Fig. 4A, B). SHMT2 decreased, while SHMT2α increased stem cell like features of ovarian cancer cells, as identified by spheroid formation ability (Fig. 4C, D), expression of stemness markers CD44 and CD133 (Fig. 4E). Furthermore, in vivo tumorigenesis of SKOV3/DDP cells was also significantly decreased by SHMT2 restored, while increased by SHMT2α restored (Fig. 4F). An online tool for extreme limiting dilution analysis (http://bioinf.wehi.edu.au/software/elda) predicted that a lower bound of 1/480652 for the frequency of repopulating control SKOV3/DDP cells, while SHMT2 restored cells were estimated to have a 1/2155647 lower repopulating cell frequency, SHMT2α restored cells were estimated to have a 1/48066 lower repopulating cell frequency (Table 1). Collectively, these results strongly demonstrated that the restored expression of SHMT2α upregulated the stem cell properties of cisplatin-resistant ovarian cells.

**Table 1:** Stem cell frequency.

Group	Lower	Estimate	Upper
Empty	480652	108183	24350
SHMT2α	48066	10819	2435
SHMT2	2155647	536021	133287

### Untargeted metabolomics shown different metabolic features between SHMT2α and SHMT2 restored ovarian cancer cells

**Fig. 5:**
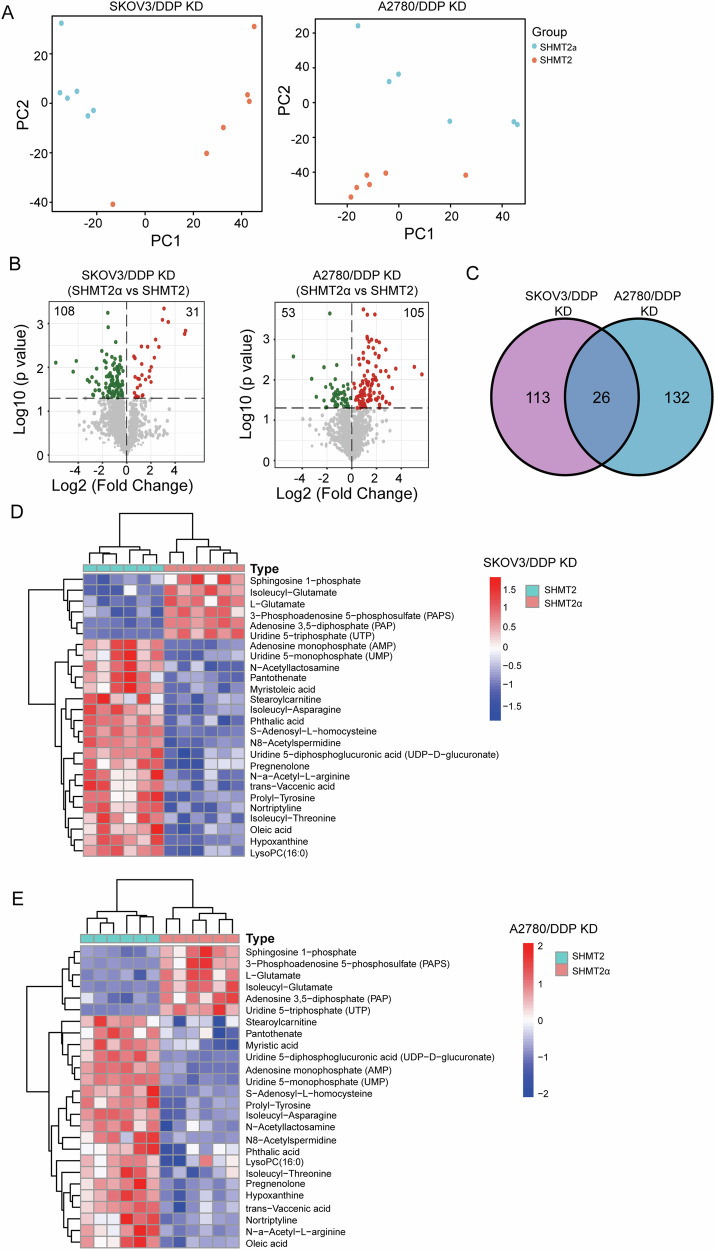
Untargeted metabolomics shown different Metabolic Features between SHMT2α and SHMT2 restored ovarian cancer cells. **A** The PCA models show the metabolic differences for different groups. **B** Volcano plots of the metabolomics with significant changes between the restored expression of SHMT2 and SHMT2α in SHMT2 knockout SKOV3/DDP and A2780/DDP cells. **C** A Venn diagram illustrating the simultaneously changed metabolites in SHMT2 or SHMT2α restored SHMT2 knockout SKOV3/DDP and A2780/DDP cells. **D**, **E** A heatmap of the differentially metabolites in SHMT2 or SHMT2α restored SHMT2 knockout SKOV3/DDP and A2780/DDP cells.

We performed metabolic sequencing analysis after restored expression of SHMT2 or SHMT2α in SHMT2 knockout SKOV3/DDP and A2780/DDP cells. The PCA analysis and volcano plots shown that there were significant metabolic differences between the restored expression of SHMT2 and SHMT2α in SHMT2 knockout SKOV3/DDP and A2780/DDP cells (Fig. 5A, B). Venn diagram illustrated there were 26 simultaneously changed metabolites in restored expression of SHMT2 or SHMT2α cells (Fig. 5C), in which majority changed metabolites were various types of amino acids (Fig. 5D, E). This might be related that SHMT2 played a crucial role in One-carbon metabolism [16].

### Selective utilization of alternative promoters of *SHMT2* in cisplatin-resistant ovarian cancer cells under metabolic stress

**Fig. 6:**
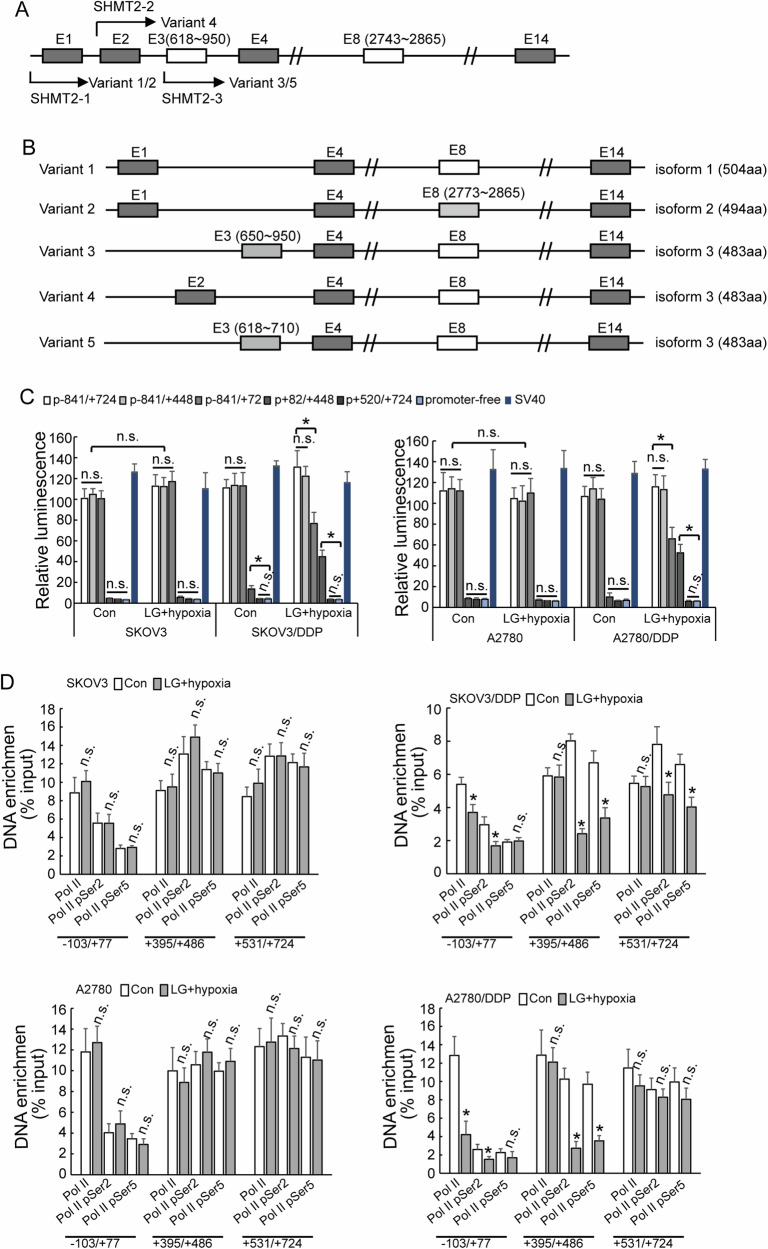
Selective utilization of alternative promoters of *SHMT2* in cisplatin-resistant ovarian cancer cells under metabolic stress. **A**, **B** Diagram of SHMT2 isoform formation. **C** Luciferase assay different promoter activity in cisplatin-sensitive (parental) and cisplatin-resistant (DDP) SKOV3 or A2780 cells under different cell culture. **D** RT-PCR shown enrichment of different RNA polymerase in cisplatin-sensitive (parental) and cisplatin-resistant (DDP) SKOV3 or A2780 cells under different cell culture.

SHMT2 gene contains three promoters, and different variants will be transcribed due to selective utilization of different promoters (Fig. 6A, B). The reporters containing −841/+72, −841/+448, −841/+724 fragments showed highest and similar activities, while no obvious activity of reporter containing +82/+448 or +520/+724 fragments was observed in both cisplatin-sensitive and cisplatin-resistant ovarian cancer cells under normal condition (Fig. 6C), indicating that ovarian cancer cells preferentially utilized promoter 1, while promoter 2 and promoter 3 were rarely activated under normal culture condition. The activity of reporter containing −841/+72 fragment significantly decreased, while the activity of reporter containing +82/+448 fragment significantly increased in SKOV3/DDP and A2780/DDP under hypoglycemic and hypoxic condition (Fig. 6C). The enrichment of RNA Pol II pSer2 and Pol II pSer5 of SKOV3/DDP and A2780/DDP to the promoter 1 region was significantly decreased under the low-glucose and hypoxic condition, while there was no significant difference in SKOV3 and A2780 cells (Fig. 6D).

### Selective utilization of *SHMT2* promoter 2 by HIF1α and TFE3 complex

**Fig. 7:**
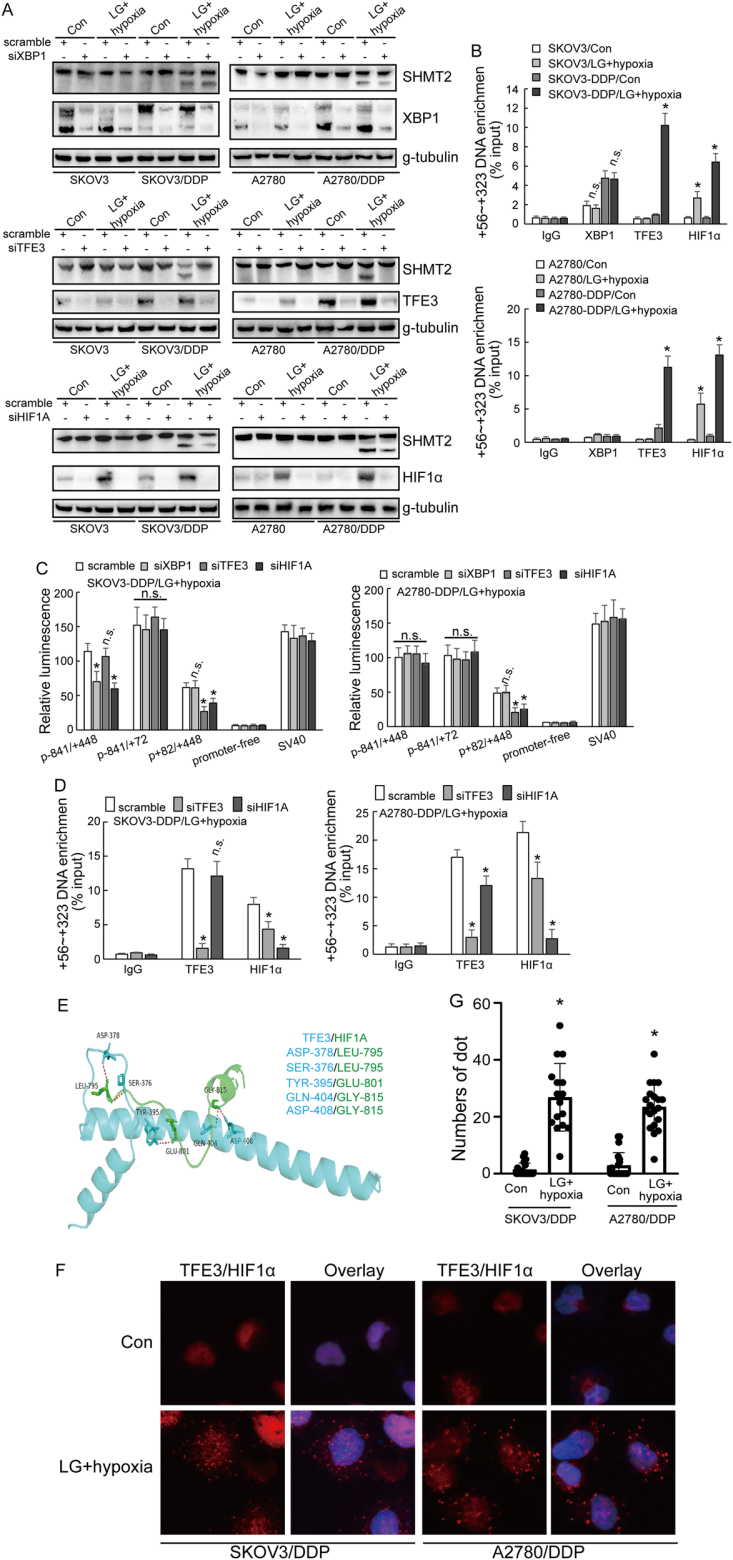
Selective utilization of *SHMT2* promoter 2 by HIF1α and TFE3 complex. **A**, **B** Western blot analysis of SHMT2, SHMT2α, XBP1, TFE3, HIF1α protein expression upon XBP1, TFE3, HIF1α knockdown respectively in cisplatin-sensitive (parental) and cisplatin-resistant (DDP) SKOV3 or A2780 cells under different cell culture. **C** Luciferase assay different promoter activity upon XBP1, TFE3, HIF1α knockdown respectively cisplatin-resistant (DDP) SKOV3 or A2780 cells under low-glucose and hypoxic environment. **D** Co-IP experiment testing the interaction between TFE3, HIF1α in cisplatin-resistant (DDP) SKOV3 or A2780 cells under low-glucose and hypoxic environment. **E** Binding pose of TFE3 with HIF1α in 3D. **F**, **G** PLA analyze showing the distribution of TFE3 with HIF1α in cisplatin-resistant (DDP) SKOV3 or A2780 cells under different cell culture.

To understand the regulation of SHMT2α by transcription factors, the specific transcription factor binding sites around promoter 2 which were associated with metabolic stress were surveyed and XBP1, HIF1α and TFE3 were screened out. Knockdown of TFE3 and HIFα in low-glucose and hypoxic condition decreased the expression of SHMT2 isoform 3, while XBP1 knockdown demonstrated no obvious effect (Fig. 7A). ChIP confirmed significant enrichment of HIF1α and TFE3 to the promoter 2 region in cisplatin-resistant cells under low-glucose and hypoxic culture condition (Fig. 7B). Knockdown of TFE3 and HIFα decreased the activity of promoter 2, and knockdown of XBP1 had no significant difference to the activity of promoter 2 (Fig. 7C). To further study whether there was a regulation between TFE3 and HIFα, ChIP showed that TFE3 knockdown inhibited the recruitment of HIF1α in both SKOV3/DDP and A2780/DDP cells (Fig. 7D). HIF1α knockdown decreased recruitment of TFE3 in A2780/DDP, while had no effect in SKOV3/DDP (Fig. 7D). Molecular docking model predicted direct interaction of HIF1α and TFE3 (Fig. 7E). PLA analyze confirmed the enhanced interaction of TFE3 and HIF1α in cisplatin-resistant ovarian cancer cells under the low-glucose and hypoxic condition (Fig. 7F, G).

## Discussion

In 1955 Thomlinson first proposed the concept of tumor hypoxia, more than 60 years of clinical and experimental evidence have shown that hypoxia is a common feature of many types of solid tumors [16]. As the tumor continues to proliferate, low glucose that often occur at the same time as hypoxia is also a common change in the tumor microenvironment (TME). This is because cancer cells are characterized by a high proliferation rate and active metabolism, and they need to consume a lot of energy to support their increased proliferation and growth rate compared to normal cells [17]. When the oxygen and glucose required by cell metabolism cannot be satisfied by organism, it will change TME into a hypoxia and low-glucose situation [18], and it might cause a series of metabolic disorders [19].

SHMT2 is a key mitochondrial enzyme in serine catabolism that converts serine to glycine [20, 21]. Studies have shown that high expression of SHMT2 may promote tumor proliferation in bladder cancer, rhabdomyosarcoma, esophageal cancer and diffuse large B-cell lymphoma [14, 22–24]. SHMT2 induces stemness of head and neck cancer, Burkitt lymphoma [25, 26]. On the contrary, SHMT2 functions as a tumor suppressor and negatively regulates proliferation and metastasis in prostate cancer [27]. These data indicate that SHMT2 might function as both tumor-promoting and tumor-suppressive functions in a cell-dependent context. The current study demonstrated that SHMT2 isoforms might exert paradoxical functions in ovarian cancer cells. SHMT2 isoform 1 might function as tumor suppressive function, as it suppressed cancer stem cell (CSC)-like features including increase of cisplatin responsiveness of parent ovarian cancer cells, suppression of spheroid formation, downregulation of CSC markers. On the contrary, SHMT2 isoforms 3 might function as tumor promoting function to promote CSC-like features. However, exact mechanisms underlying discrete roles of SHMT2 isoforms in ovarian cancer require further investigation. Anyway, the current study suggested that discrete isoform expression might represent another layer of paradoxical function of SHMT2 in different cancer.

In order to survive under metabolic stress, cancer cells regulate metabolism, protein synthesis and cell cycle processes by the adjustment of transcription factors [28, 29]. Hypoxia is a common characteristic of the tumor microenvironment found in most solid tumors, including ovarian cancer [30]. Cancer cells under hypoxic stress regulate protein translation through reprogramming gene expression, leading to microenvironmental changes [31]. HIF1-α is an important regulatory transcription factor in the hypoxia and low-glucose situation. Accumulating data indicate complex implication of SHMT2 in hypoxia. Co-induction of SHMT2 by HIF1α and Myc maintains cell growth by balancing the NADPH/NADP^+^ ratios in neuroblastoma tumors upon lack of oxygen [32]. Hypoxia stabilizes SHMT2 lactation, thereby promoting glycolysis and stemness of esophageal cancer cells [24]. Furthermore, SHMT2 regulates HIF1α expression through both enzymatic and non-enzymatic functions in gastric cancer [33]. Identification of selective utilization of SHMT2 alternative promoter by the combination of HIFα and TFE3 in cisplatin-resistant ovarian cancer cells in the current study further strengthen the complicated interlinkage between hypoxia and SHMT2.

Many genes have multiple promoters, which may even lead to different functions of the same gene. The utilization of alternative promoter produces different pre-mRNAs being transcribed from variable transcription start sites (TSSs) within a gene locus. The patterns of alternative promoter selection result in the regulation of diverse cell types, tissue types and complex developmental genes. The abnormal usage of alternative promoter plays an important role in the occurrence and development of various diseases. As a general mechanism, alternative promoter is diverse and flexible in gene expression regulation. In the current study, we reported that under hypoxia and hypoglycemia, the combination of HIFα and TFE3 leads to a reduction in the utilization of promoter 1, while the increase in the selection of promoter 2, resulting in transcriptional dysregulation and an increase in the transcription of variant 4, followed by increase of SHMT2 isoform 3 under metabolic stress conditions. In general, the high expression of SHMT2α and the low expression of SHMT2 in the hypoxia and low-glucose situation may be one of the causes of cisplatin resistance in ovarian cancer patients. However, further studies are needed to dissect, how SHMT2 isoforms exerted paradoxical functions in ovarian cancer.

Collectively, the current study demonstrated that ovarian cancer cells expressed multiple SHMT2 isoforms, which exerted complicated functions. Selective utilization of SHMT2 alternative promoter by HIF1α and TFE3 resulted in SHMT2 isoform expression shift, which might be implicated in adaptation of ovarian cancer cells under metabolic stress.

## Supplementary information

Original WB

## Data Availability

The datasets generated during and/or analyzed during the current study are available from the corresponding author on reasonable request.
