# Supplementary figures and images for: TFE3 and HIF1α regulates the expression of SHMT2 isoforms via alternative promoter utilization in ovarian cancer cells
Source: Cell Death Dis. 2025 Mar 17;16(1):178. doi: 10.1038/s41419-025-07445-y (PMC11914208; doi:10.1038/s41419-025-07445-y)

Fig1

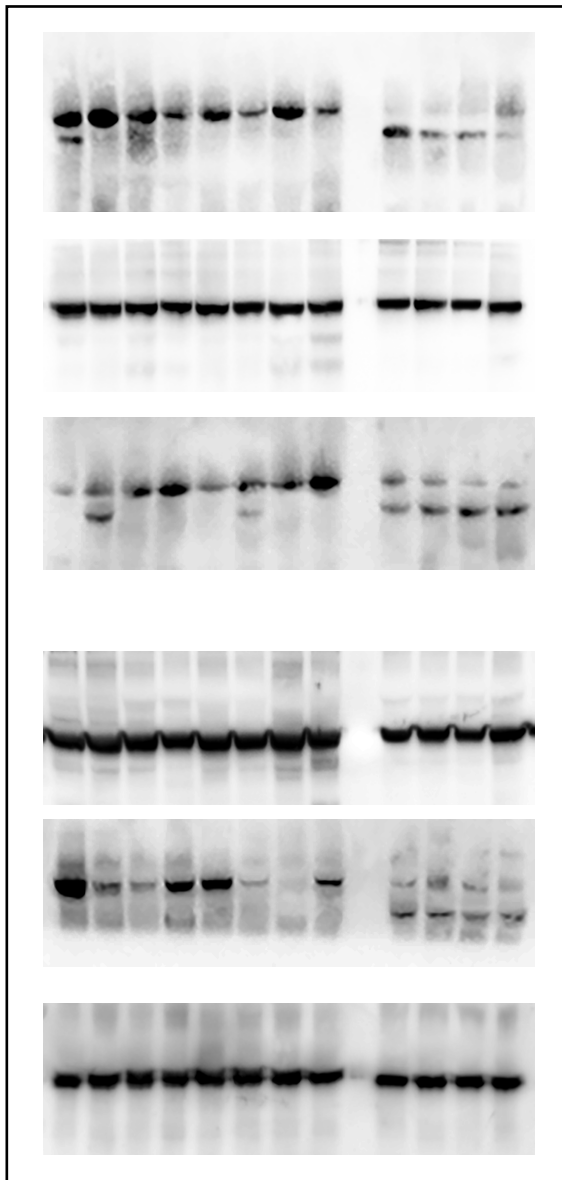

Fig2

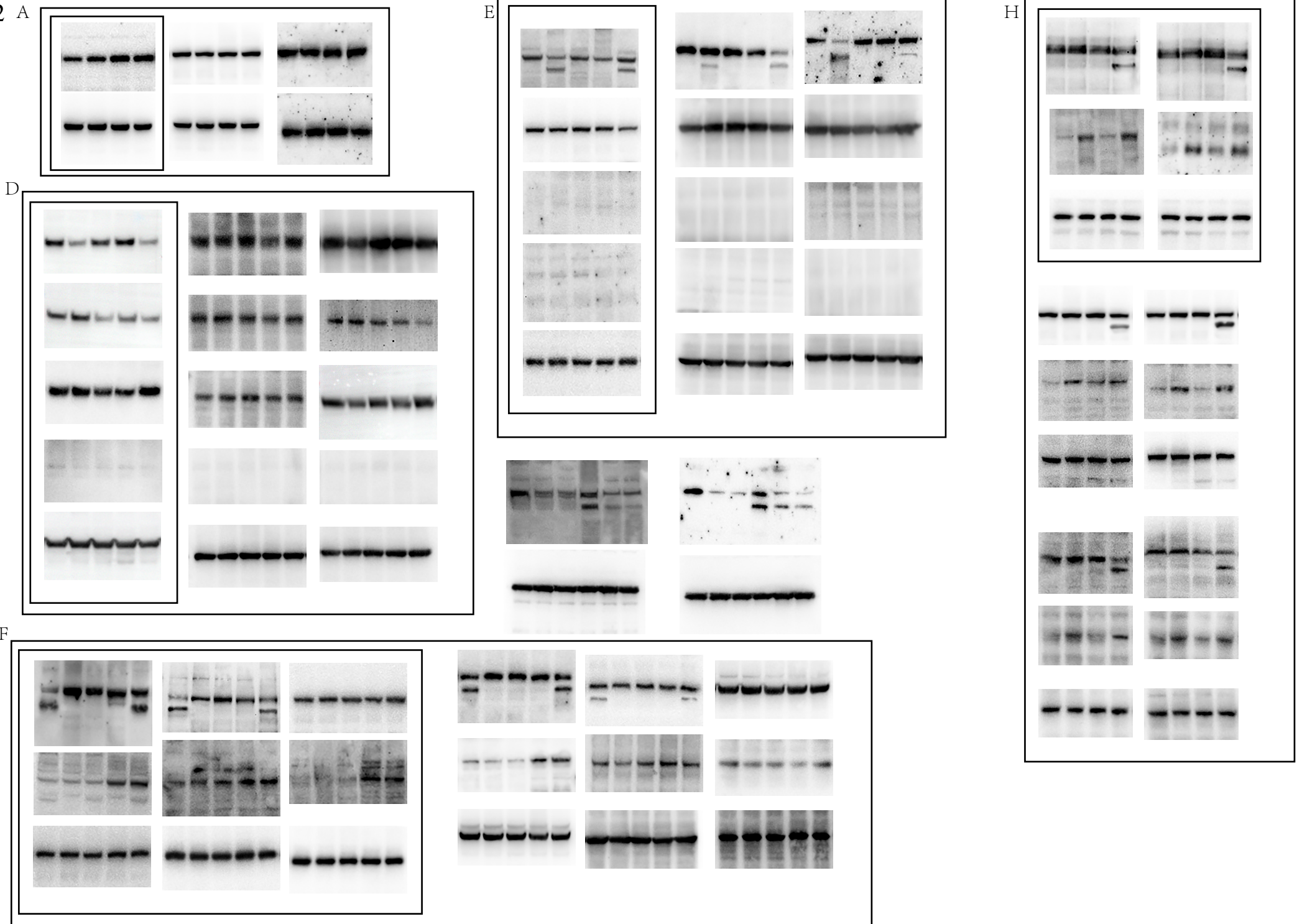

Fig3

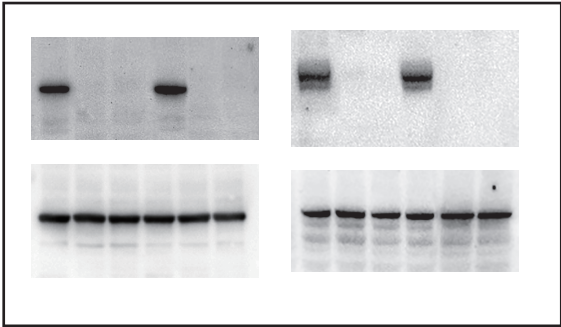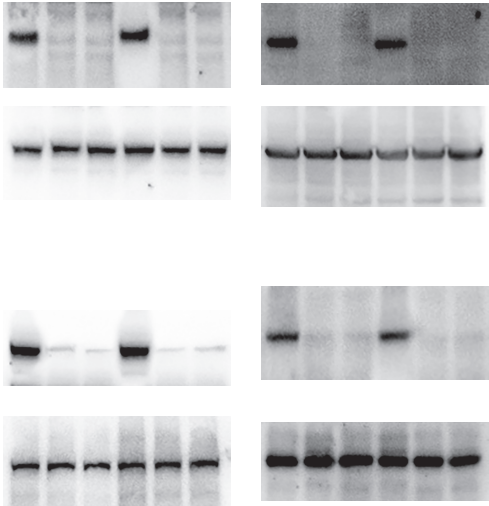

Fig4

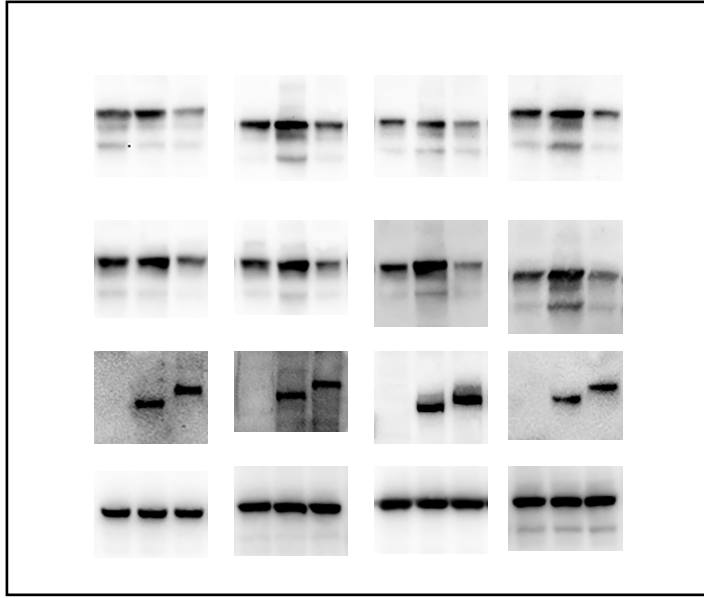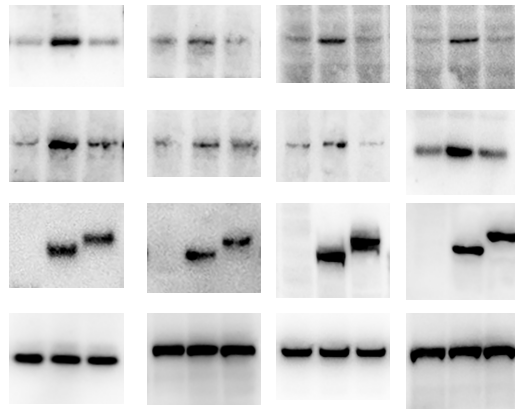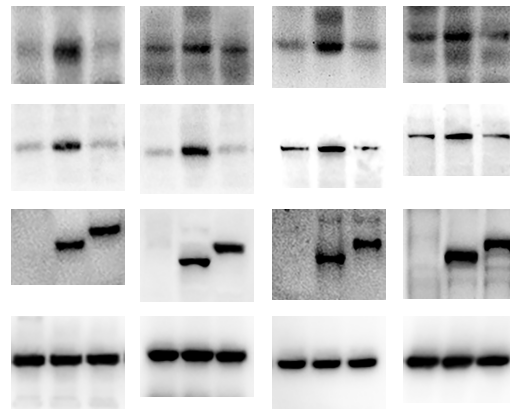

Fig 7

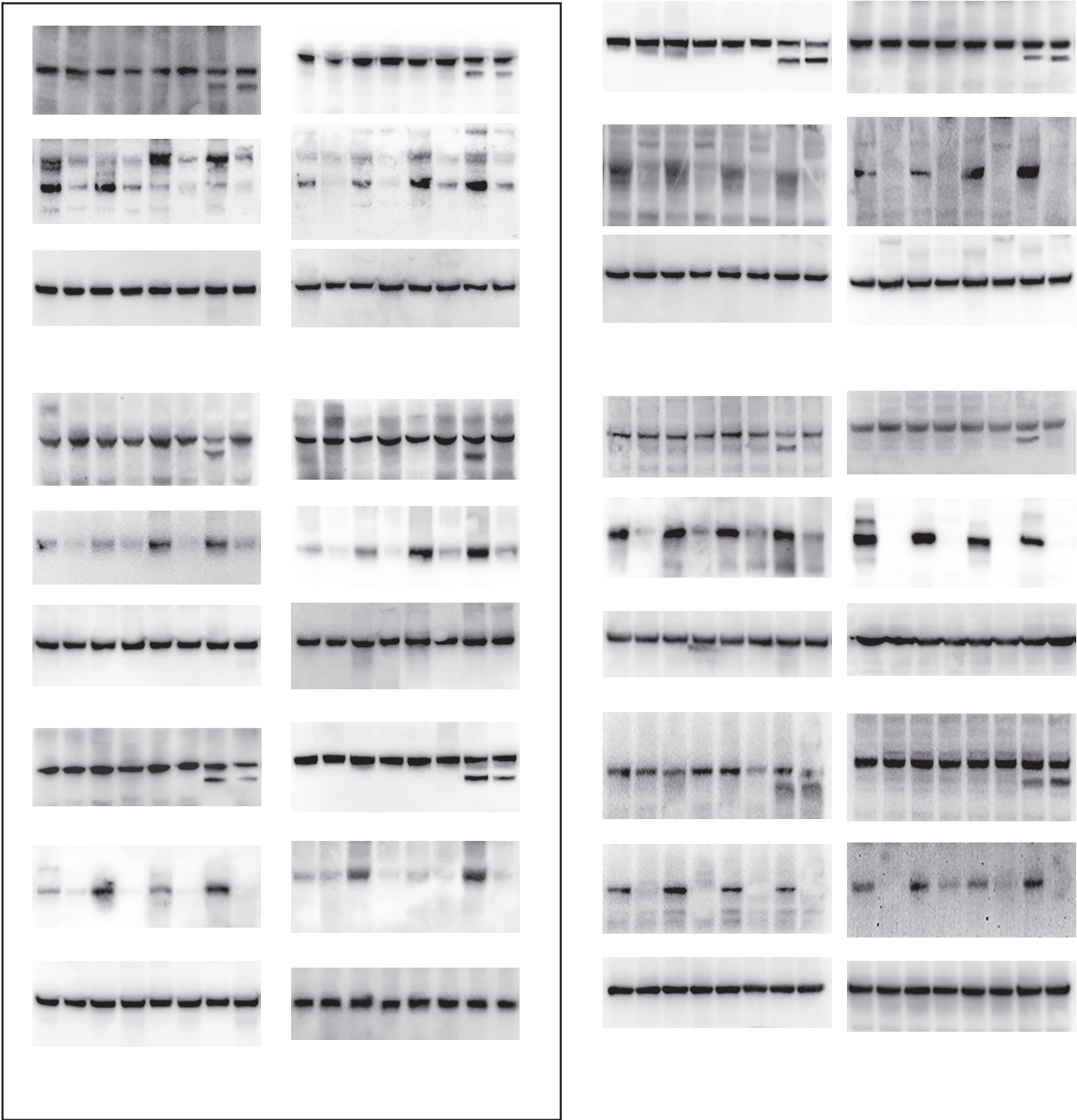

Supplement: Supplementary file 1 — Original WB [file 41419_2025_7445_MOESM1_ESM.pdf]
